# Substrate reduction therapy for Krabbe disease and metachromatic leukodystrophy using a novel ceramide galactosyltransferase inhibitor

**DOI:** 10.1038/s41598-021-93601-1

**Published:** 2021-07-14

**Authors:** Michael C. Babcock, Christina R. Mikulka, Bing Wang, Sanjay Chandriani, Sundeep Chandra, Yue Xu, Katherine Webster, Ying Feng, Hemanth R. Nelvagal, Alex Giaramita, Bryan K. Yip, Melanie Lo, Xuntian Jiang, Qi Chao, Josh C. Woloszynek, Yuqiao Shen, Shripad Bhagwat, Mark S. Sands, Brett E. Crawford

**Affiliations:** 1 BioMarin Pharmaceutical Inc., 105 Digital Drive, Novato, CA 94949 USA; 2 Department of Medicine, Washington University School of Medicine, St. Louis, MO 63110 USA; 3 Department of Genetics, Washington University School of Medicine, St. Louis, MO 63110 USA; 4 Department of Pediatrics, Washington University School of Medicine, St. Louis, MO 63110 USA

**Keywords:** Glycobiology, Diseases of the nervous system, Target validation, Neurological disorders

## Abstract

Krabbe disease (KD) and metachromatic leukodystrophy (MLD) are caused by accumulation of the glycolipids galactosylceramide (GalCer) and sulfatide and their toxic metabolites psychosine and lysosulfatide, respectively. We discovered a potent and selective small molecule inhibitor (S202) of ceramide galactosyltransferase (CGT), the key enzyme for GalCer biosynthesis, and characterized its use as substrate reduction therapy (SRT). Treating a KD mouse model with S202 dose-dependently reduced GalCer and psychosine in the central (CNS) and peripheral (PNS) nervous systems and significantly increased lifespan. Similarly, treating an MLD mouse model decreased sulfatides and lysosulfatide levels. Interestingly, lower doses of S202 partially inhibited CGT and selectively reduced synthesis of non-hydroxylated forms of GalCer and sulfatide, which appear to be the primary source of psychosine and lysosulfatide. Higher doses of S202 more completely inhibited CGT and reduced the levels of both non-hydroxylated and hydroxylated forms of GalCer and sulfatide. Despite the significant benefits observed in murine models of KD and MLD, chronic CGT inhibition negatively impacted both the CNS and PNS of wild-type mice. Therefore, further studies are necessary to elucidate the full therapeutic potential of CGT inhibition.

## Introduction

**Figure 1:**
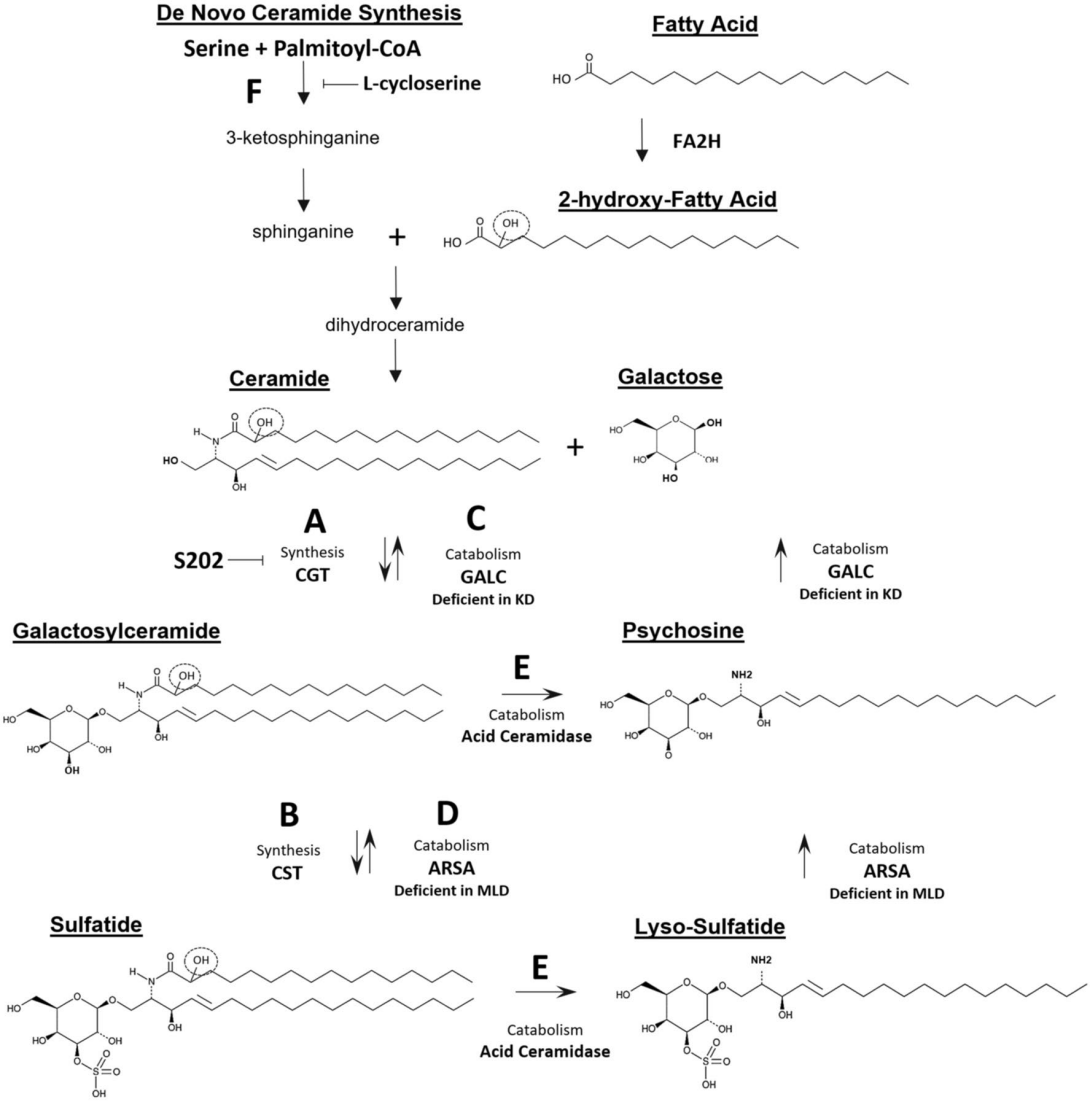
Synthesis and catabolism of galactosylceramide and sulfatide. CGT catalyzes the synthesis of GalCer by adding a galactose molecule to ceramide (**A**). Ceramide with a hydroxy group at the 2 position (dashed circle) is a more efficient CGT substrate than non-hydroxyceramide. CST catalyzes the sulfation of GalCer to generate both non-hydroxy and 2-hydroxy-sulfatide (**B**). GalCer and sulfatide are degraded in the lysosome by the enzymes GALC and ARSA, which are deficient in KD and MLD, respectively (**C,D**). GALC and ARSA deficiency results in lysosomal accumulation of these glycolipids and their subsequent deacylation into the toxic byproducts psychosine and lysosulfatide by acid ceramidase (**E**). Previous substrate reduction approaches using L-cycloserine targets a reaction upstream from ceramide synthesis (**F**).

Krabbe disease (KD) and metachromatic leukodystrophy (MLD) are severe, dysmyelinating or demyelinating diseases of the peripheral and central nervous systems (PNS and CNS)^1,2^. The infantile forms are characterized by developmental delay, loss of milestones, motor dysfunction, and death by 2–4 years of age. Krabbe disease and MLD are caused by deficiencies in the lysosomal enzymes galactosylceramidase (GALC) and arylsulfatase A (ARSA), respectively. Together, these enzymes catalyze the degradation of major glycosphingolipids of the myelin sheath—galactosylceramide and sulfatide (3-O sulfated galactosylceramide) (Fig. 1). In addition, these enzymes degrade the highly toxic, deacylated byproducts of GalCer and sulfatide metabolism, namely galactosylsphingosine (psychosine) and 3-O sulfogalactosylsphingosine (lysosulfatide), respectively. In the absence of GALC or ARSA these toxic metabolites accumulate leading to profound demyelination. In the case of KD, it has been shown that the accumulation of psychosine is responsible for most of the acute pathology and clinical signs^3^.

UDP-galactose:ceramide galactosyltransferase (CGT, EC 2.4.1.45), encoded by the *UGT8* gene, catalyzes the addition of galactose from UDP-galactose to ceramide to generate GalCer (Fig. 1A). Ceramide galactosyltransferase can utilize either 2-hydroxylated or non-hydroxylated ceramides as a substrate; however, 2-hydroxylated ceramides have been shown to be more efficiently galactosylated compared to non-hydroxylated forms^4,5^. While CGT can generate low levels of GalCer in a variety of cell types, the enzyme is most highly expressed in oligodendrocytes and Schwann cells during periods of active myelination^6^. GalCers and sulfatides make up > 25% of the lipids in mature myelin membranes with GalCer being more abundant than sulfatides^6^. During myelin formation, the 2-hydroxylated and non-hydroxylated forms of these molecules are synthesized in near equal proportions. Genetic studies using CGT knockout mice revealed GalCer synthesis is required to produce stable myelin in the CNS and PNS and absence of CGT results in early demyelinating disease, neurological dysfunction, and death^7–9^. It has been further suggested that the hydroxylated versions of these molecules (2-hydroxy-GalCer and 2-hydroxy-sulfatide) play specific roles in stabilizing tightly packed myelin membranes^10–12^. Consistent with this hypothesis, rodents and humans deficient in the fatty acid 2-hydroxylase develop leukodystrophy^13,14^.

The discovery of small molecules that inhibit the synthesis of galactosylceramide-based glycolipids that accumulate in KD and MLD is an area of active research^15^. Studies using small molecule glucosylceramide synthase inhibitors to reduce the abundance of the glycolipids that accumulate in Gaucher disease, Fabry disease, GM1 gangliosidosis, and GM2 gangliosidosis, have demonstrated the benefits of substrate reduction therapy^16–20^. Data from mice that are completely deficient in both CGT and GALC suggest that lowering GalCer levels by CGT inhibition provides some benefit for KD^21,22^. Partial loss of CGT activity in mice heterozygous for a *Ugt8* null allele show that reducing GalCer levels to approximately 70% of normal is tolerated and provides survival benefit to Krabbe mice^22^. However, the absence of potent and selective inhibitors of GalCer and sulfatide synthesis has impeded the development of a substrate reduction therapy (SRT) for KD or MLD. Although L-cycloserine has been used to evaluate the benefits of reducing the synthesis of galactosylceramide in GALC-deficient mice it acts several steps upstream of GalCer synthesis through inhibition of serine palmitoyltransferase and is unlikely to be used clinically due to lack of specificity and resulting toxicity (Fig. 1F)^23^. However, these studies provided proof-of-concept that partially reducing psychosine levels can extend survival and reduce pathology of Krabbe mice^24–26^. Although the therapeutic effect of L-cycloserine was modest, this approach synergized with orthogonal therapeutic strategies, including bone marrow transplant and gene therapy to greatly increase efficacy^27^.

We have discovered a novel, potent, selective, and brain-penetrant small molecule inhibitor of CGT (S202) and determined its potential as an SRT to treat KD and MLD. When administered to mice, S202 significantly decreased psychosine accumulation and increased life span of GALC-deficient Twitcher mice without overt toxicity. Similar doses of S202 reduced lysosulfatide levels in ARSA-deficient MLD mice. However, a closer examination of wild-type mice treated with S202 revealed negative impacts of CGT inhibition on myelin rich tissues including brain, spine, and peripheral nerves. In total, S202 or its analogs provide a critical step forward toward the development of a safe and efficacious SRT for KD and MLD.

## Results

### Discovery of a potent and selective small molecule inhibitor of CGT

**Figure 2:**
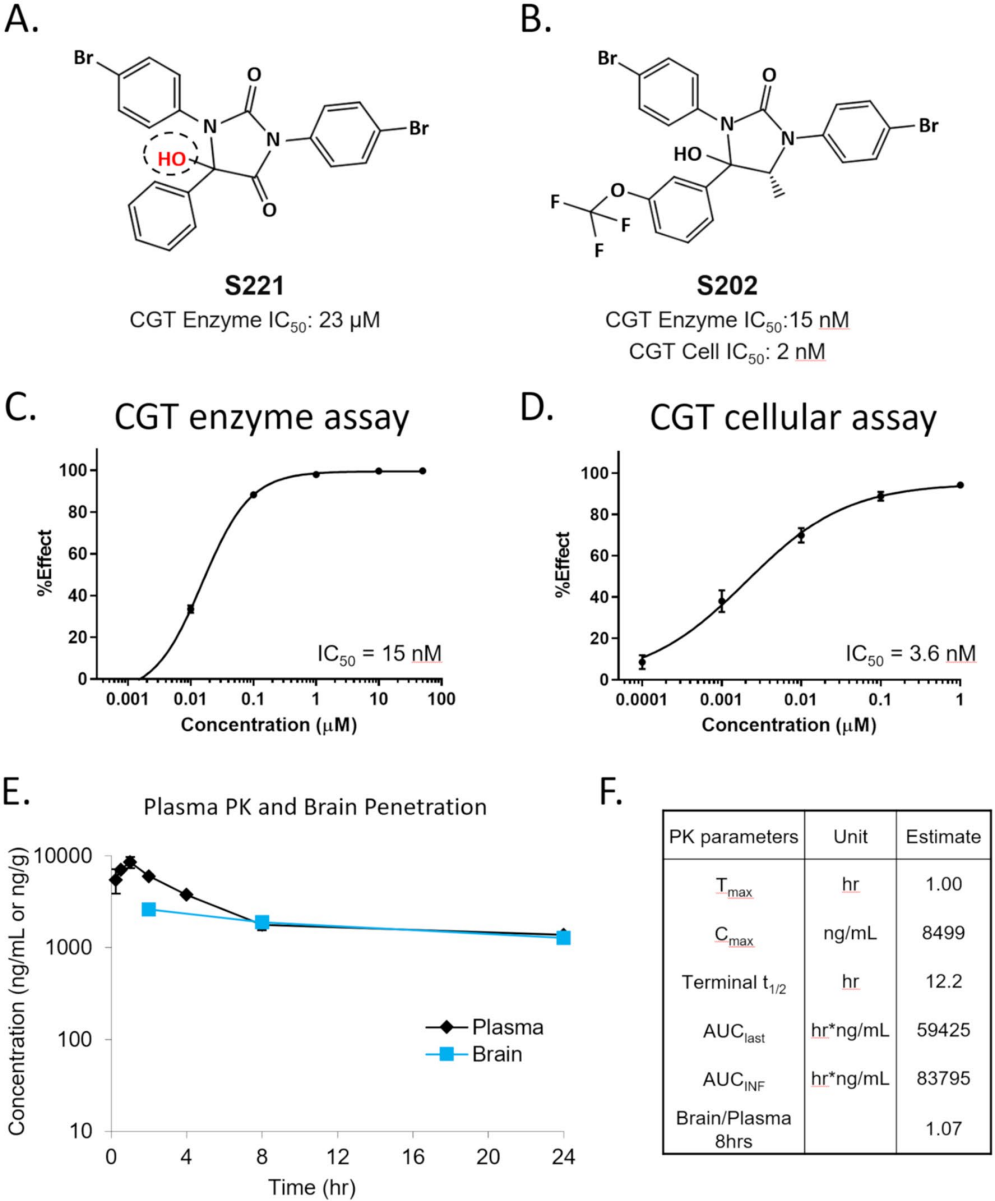
Identification of a novel potent and brain penetrant CGT inhibitor. S221 was identified in a screen of a focused bioactive lipid library (**A**). S221 was determined to be an oxidation product of (CAY10508), which had acquired a hydroxyl group (in red) on the central ring. Through a medicinal chemistry effort S202 was discovered as a more potent CGT inhibitor (**B**) showing a 400-fold increased potency in a CGT enzyme assay (**C**) and single digit nanomolar potency in a CGT cellular assay (**D**) (n = 2 per concentration). Mouse pharmacokinetic (PK) and brain concentration profile graph (n = 3, **E**) and PK parameter table (**F**) for S202 after a single IP injection show favorable in vivo half-life and brain penetration properties.

A novel CGT inhibitor was identified by screening a small, focused library of bioactive lipids for inhibitors of CGT catalytic activity. Human CGT was obtained from lysates of CHO cells expressing human *UGT8*, the gene encoding CGT. A single compound was identified from this screen that weakly inhibited CGT activity (Fig. 2A). This molecule had previously been described as a cannabinoid receptor (CB1) inverse agonist^28^. Surprisingly, after re-synthesizing this screening hit based on the described structure, the CGT inhibitory activity was lost. Subsequent LC–MS analysis of both the original and resynthesized material confirmed that these two molecules were different. After careful evaluation of the analytical data, it was determined that the active molecule contained an additional hydroxyl group on its central 5-member ring (Fig. 2A red text) and was likely an oxidation product of the original compound. When the hydroxylated version was synthesized (S221), CGT inhibition was restored with an IC_50_ of 23 µM.

Following identification of the initial low potency CGT inhibitor S221, a series of structural analogs with increased potency were synthesized^29^. One analog, hereafter referred to as S202, displayed improved potency with an IC_50_ of 15 nM in the cell-lysate assay and 3.6 nM (n = 6) in an intact-cell assay (Fig. 2B–D). Pharmacokinetic studies revealed that S202 had an in vivo half-life of 12.2 h and readily distributed into the mouse brain, achieving a brain to plasma ratio of ~ 1 after 8 h (Fig. 2E,F). Through the optimization of S221 for CGT inhibitory properties, the potency as a CB1 inverse agonist was reduced tenfold. The inhibitory activity of S202 was highly selective for CGT, with minimal to no activity toward other enzymes that act on related glycosphingolipids (see Supplementary Table S1 online).

### CGT inhibition preferentially reduces non-hydroxy-GalCer synthesis in vivo

**Figure 3:**
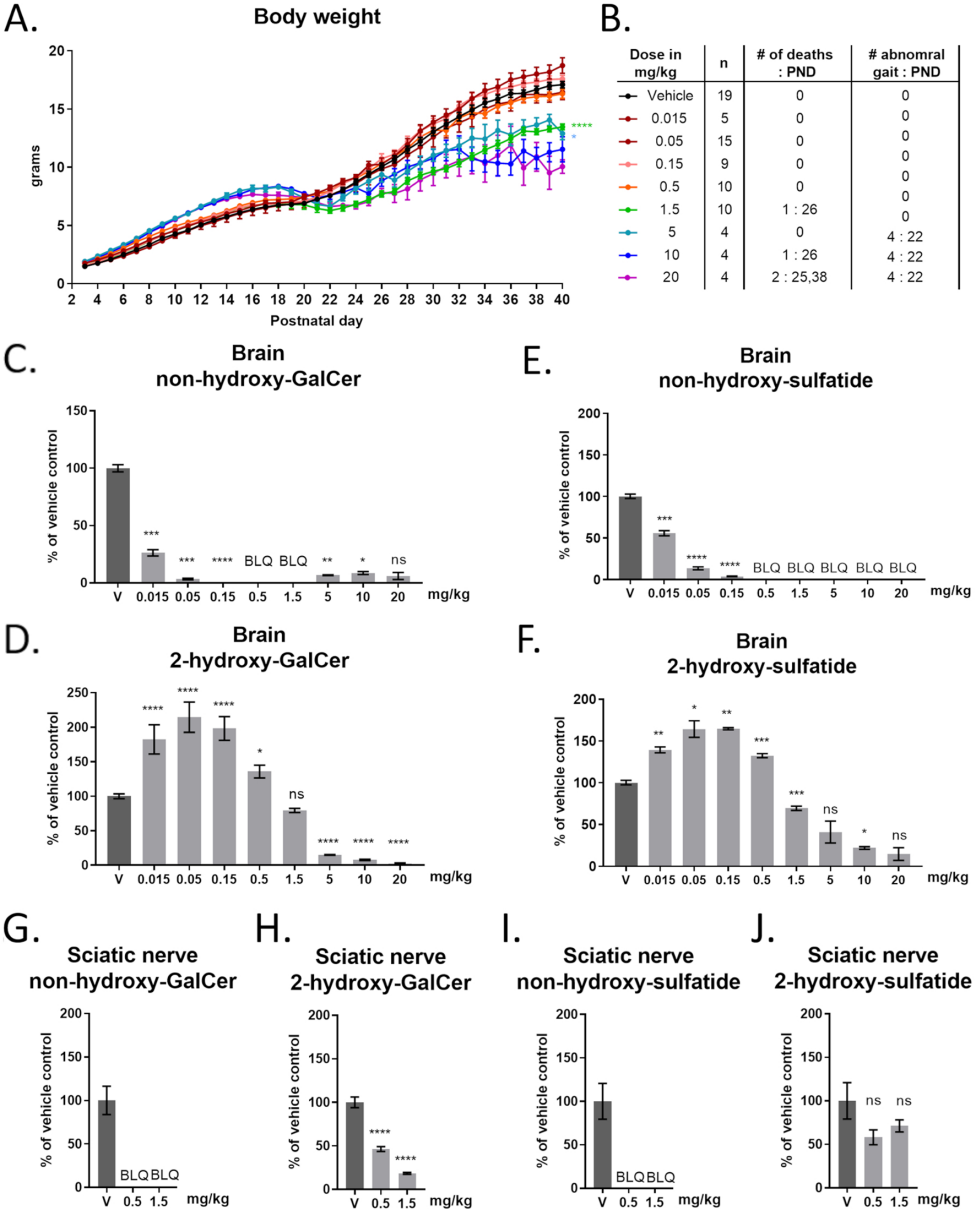
Tolerability and biochemical changes in young mice treated with S202. Weight profiles of neonatal mice (n = 4–19) treated with daily IP S202 (mg/kg/day) from PND3 to PND40 (**A**) and occurrence of animal deaths and development of abnormal gait (**B**). LC–MS quantification of brain non-hydroxy-GalCer (**C**), 2-hydroxy-GalCer (**D**), non-hydroxy-sulfatide (**E**), and 2-hydroxy-sulfatide (**F**) from mice treated with S202. Sciatic nerve non-hydroxy-GalCer (**G**), 2-hydroxy-GalCer (**H**), non-hydroxy-sulfatide (**I**), and 2-hydroxy-sulfatide (**J**) from mice treated with S202. Refer to the Materials and Methods section for significance levels.

Ceramide galatosyltransferase mRNA expression peaks in mice during periods of major myelin synthesis, corresponding roughly to postnatal days (PND) 10–30 for the CNS and PND 6–15 for the PNS^6,30^. Large quantities of galactosylceramides and sulfatides are incorporated into mature myelin with half-lives approaching 1 year^31^. Therefore, to evaluate the effects of CGT inhibition, wild-type mice received daily intraperitoneal (IP) injections of S202 starting on PND3, before abundant synthesis of these glycolipids have occurred. Treatment continued until PND40, after the majority of myelination is complete in both the CNS and PNS. S202 was well tolerated at lower doses; however, above 0.5 mg/kg produced phenotypes that were strikingly similar to those reported in the CGT knockout mouse including reduced weight gain, mild hind limb gait abnormalities, and some animal deaths (Fig. 3A,B)^21^.

GalCer and sulfatide levels in S202-treated mice were monitored by LC–MS/MS. Lower doses (0.015 to 0.5 mg/kg) of S202 greatly decreased non-hydroxy-GalCer levels in the brains of wild-type mice without reducing 2-hydroxy-GalCer (Fig. 3C,D). Remarkably, lower doses of S202 were found to consistently increase 2-hydroxy-GalCer levels. However, higher doses of S202 (> 1.5 mg/kg), reduced synthesis of both non-hydroxy-GalCer and 2-hydroxy-GalCer. Like their respective GalCer precursors, treatment with lower doses of S202 selectively reduced non-hydroxy-sulfatide and correspondingly increased 2-hydroxy-sulfatide, while higher doses reduced total sulfatide levels (Fig. 3E,F). While these data represent the sum response of all acyl chain lengths, a similar response was seen when individual acyl chains were monitored (see Supplementary Fig. S1 online). Preferential reduction in non-hydroxy-GalCer and non-hydroxy-sulfatides relative to the hydroxylated versions were also seen in the sciatic nerve. However, the effect at a given dose appeared greater in the sciatic nerve relative to the brain. For example, a dose of 0.5 mg/kg increased 2-hydroxy GalCer and 2-hydroxy sulfatide in the brain while partially reducing these molecules in the sciatic nerve (Fig. 3G–J).

### CGT inhibition extends survival and improves pathology in a KD mouse model

**Figure 4:**
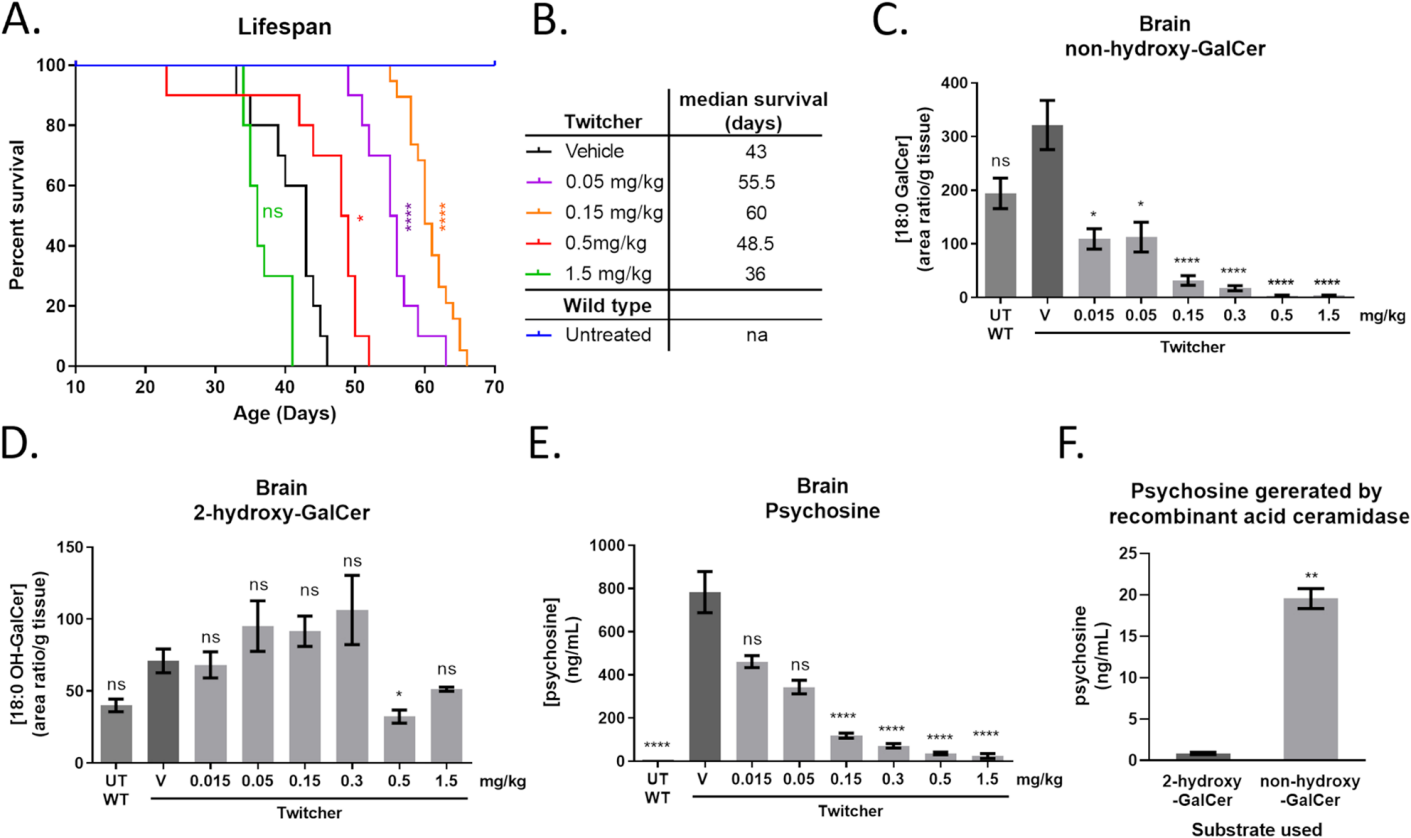
CGT inhibition extends survival and reduces psychosine levels in a KD mouse model. Survival curves of Twitcher mice treated with S202 injected IP three times per week starting on PDN3 (**A**) and median days of survival (**B**). Untreated wild-type (UT WT) mice survived while vehicle treated Twitcher mice (vehicle) had a median survival of 43 days. S202-treated Twitcher mice survived longer except at the highest dose tested (1.5 mg/kg). Effect of S202 treatment on non-hydroxy-GalCer (**C**) and 2-hydroxy-GalCer in brain homogenates from mice treated from PND3-PND28 (**D**). Psychosine reduction in these brains correlates with reduction of non-hydroxy-GalCer (**E**) (n = 5–8, **C**–**E**). Quantification of psychosine generated by recombinant acid ceramidase incubated with either purified 2-hydroxy-GalCer or non-hydroxy-GalCer substrates (n = 2 per condition, **F**).

To evaluate the therapeutic potential of CGT inhibition in a relevant disease model, we treated Twitcher mice with S202. Mice received IP injections of S202 three times per week starting on PND3 and disease progression was monitored by the onset of clinical signs and survival. The median lifespan of untreated or vehicle treated Twitcher mice is ~ 40 days^32,33^. Lower doses of S202 increased median survival to 60 days at an optimal dose of 0.15 mg/kg IP three times per week (Fig. 4A,B). However, higher doses of S202 were less effective and the highest dose tested (1.5 mg/kg) reduced lifespan.

A separate cohort of Twitcher mice was dosed and sacrificed at PND28 to evaluate the biochemical and histological effects of CGT inhibition. As observed in wild-type animals, S202 produced a dose-dependent reduction in non-hydroxy-GalCer without reducing 2-hydroxy-GalCer in the brain (Fig. 4C,D). Surprisingly, psychosine levels were reduced to the same extent as the non-hydroxy-GalCer, even though 2-hydroxy-GalCer was not reduced (Fig. 4E).

To investigate the mechanism for this correlation between psychosine and non-hydroxy-GalCer but not 2-hydroxy-GalCer, we examined the ability of recombinant acid ceramidase to deacylate non-hydroxy-GalCer and 2-hydroxy-GalCer in vitro. We recently showed that psychosine is produced through deacylation of GalCer by acid ceramidase, rather than addition of galactose to sphingosine^3^. However, it was not clear if acid ceramidase preferred either form of GalCer. Consistent with our in vivo data, acid ceramidase was found to deacylate non-hydroxy-GalCer much more efficiently than 2-hydroxy-GalCer (Fig. 4F). This important finding extends the mechanistic understanding of the origins of psychosine in Krabbe disease, indicating that psychosine is generated specifically from deacylation of non-hydroxyl-GalCer but not hydroxylated Gal-Cer.

**Figure 5:**
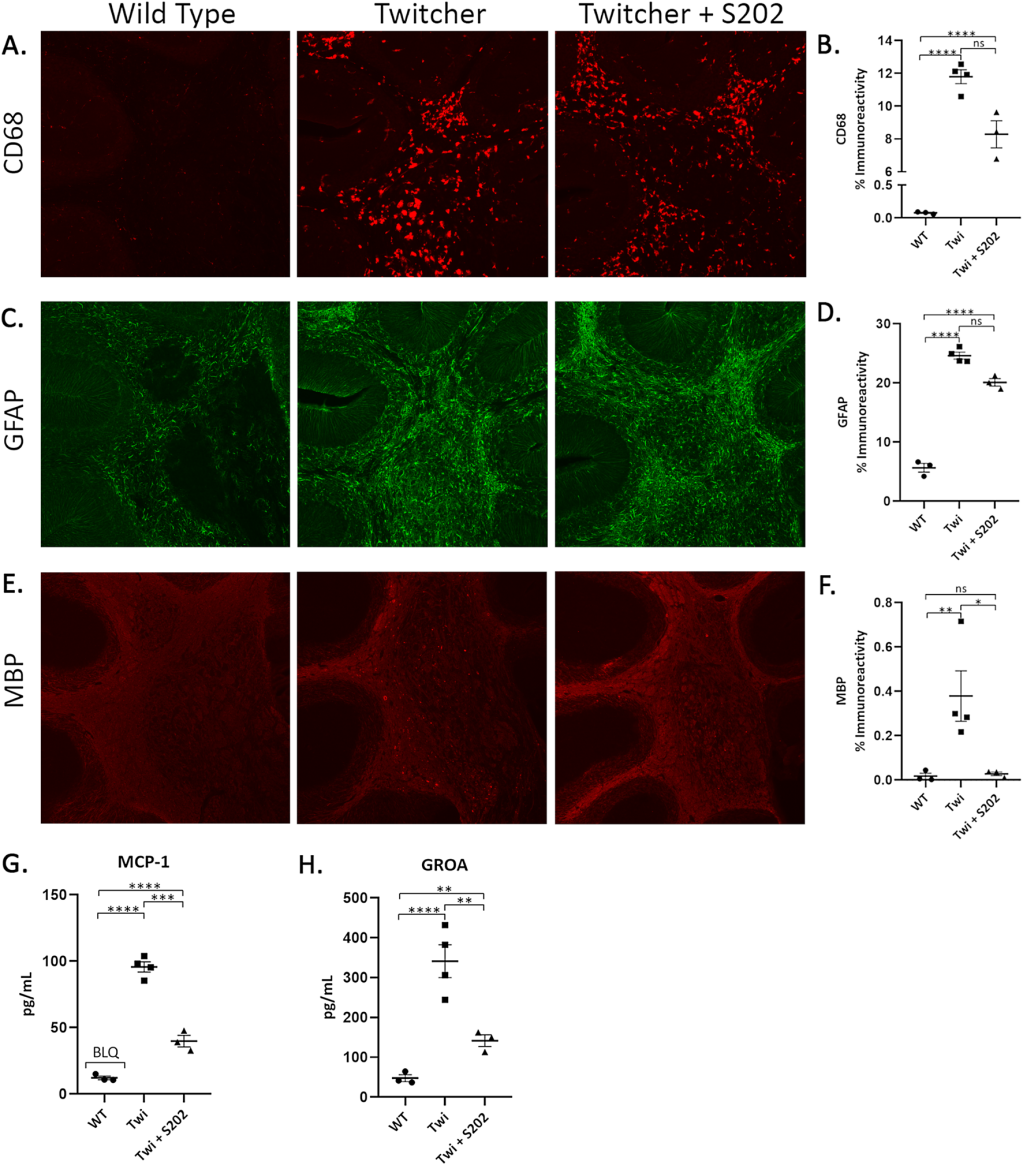
CGT inhibition improves histological and immunological markers in Twitcher mice. Brain sections from Twitcher (Twi) mice treated with 0.15 mg/kg S202 by IP injection three times per week from PND3 to PND28 were immunologically stained for CD68, GFAP and MBP (**A,C,E**) and quantified (**B,D,F**). Percent immunoreactivity represents the area of staining within the region analyzed (cerebellar white matter, 100%). Cytokine levels from brain homogenates harvested from the same mice were analyzed and MCP-1 and GROA were reduced upon S202 treatment (**G**,**H**). N = 3–4 (**A–H**).

The effects of CGT inhibition on brain pathology were examined by immunofluorescent staining for inflammatory markers and myelin basic protein in PND28 Twitcher mice. Treatment with 0.15 mg/kg S202 IP three times per week showed a trend toward reduction in the levels of CD68-positive macrophages (Fig. 5A,B) and a marker of astrocytosis (GFAP, Fig. 5C,D) in Twitcher mouse brain sections. A significant reduction was seen in a marker of myelin degeneration (MBP, Fig. 5E,F). Cytokine analysis was also performed on brain lysates from the same animals treated with S202. Treatment reduced the levels of the macrophage markers MCP-1 and GROA in brain homogenates (Fig. 5G,H). Twitcher mice had disorganized myelin with a relatively large number of intensely staining puncta throughout the white matter tracts of the cerebellum (Fig. 5E). This led to an overall increase in myelin staining compared to wild-type mice (Fig. 5F). The myelin in S202-treated animals remained disorganized but had an apparent decrease in the number of intensely staining puncta.

### Increased S202 tolerability in older Twitcher mice

**Figure 6:**
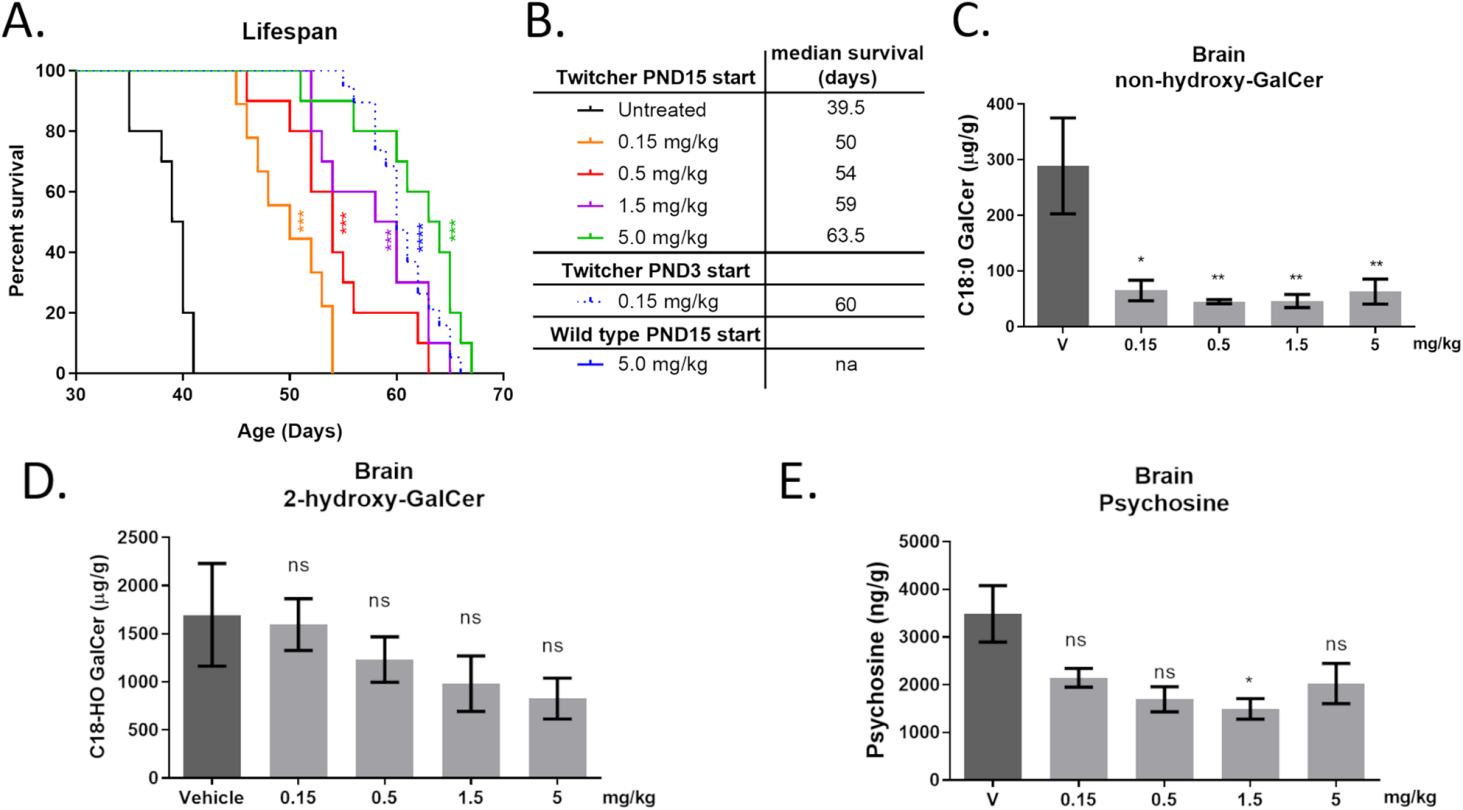
Older Twitcher mice better tolerate S202 but higher doses are required to increase survival. Survival curves of Twitcher mice treated with S202 starting on PND15 (**A**) and median days of survival (**B**). Treating Twitcher mice starting from PND15 with S202 dose dependently reduced non-hydroxy-GalCer (**C**), 2-hydroxy-GalCer (**D**), and psychosine (**E**) (n = 3–4).

To determine the effects of CGT inhibition at an age that is roughly equivalent to the myelination status of a 1 to 2-year-old human, Twitcher mice were treated with varying doses of S202 IP three times per week starting at PND15^30^. These mice showed a dose-dependent increase in survival with a maximum median life span of ~ 63 days at 5.0 mg/kg (Fig. 6A,B). Although the increase in median lifespan was comparable to that seen when treatment was initiated at PND3, an ~ 30-fold greater dose was required. Like mice treated on PND3, the increase in median survival correlated with decreases in non-hydroxy-GalCer and psychosine (Fig. 6C–E). Notably, the magnitude of the psychosine reduction was less when treatment was started later in development and only the 1.5 mg/kg dose level achieved statistical significance.

### S202 treatment reduces non-hydroxy-sulfatides and lysosulfatide in an MLD mouse model

**Figure 7:**
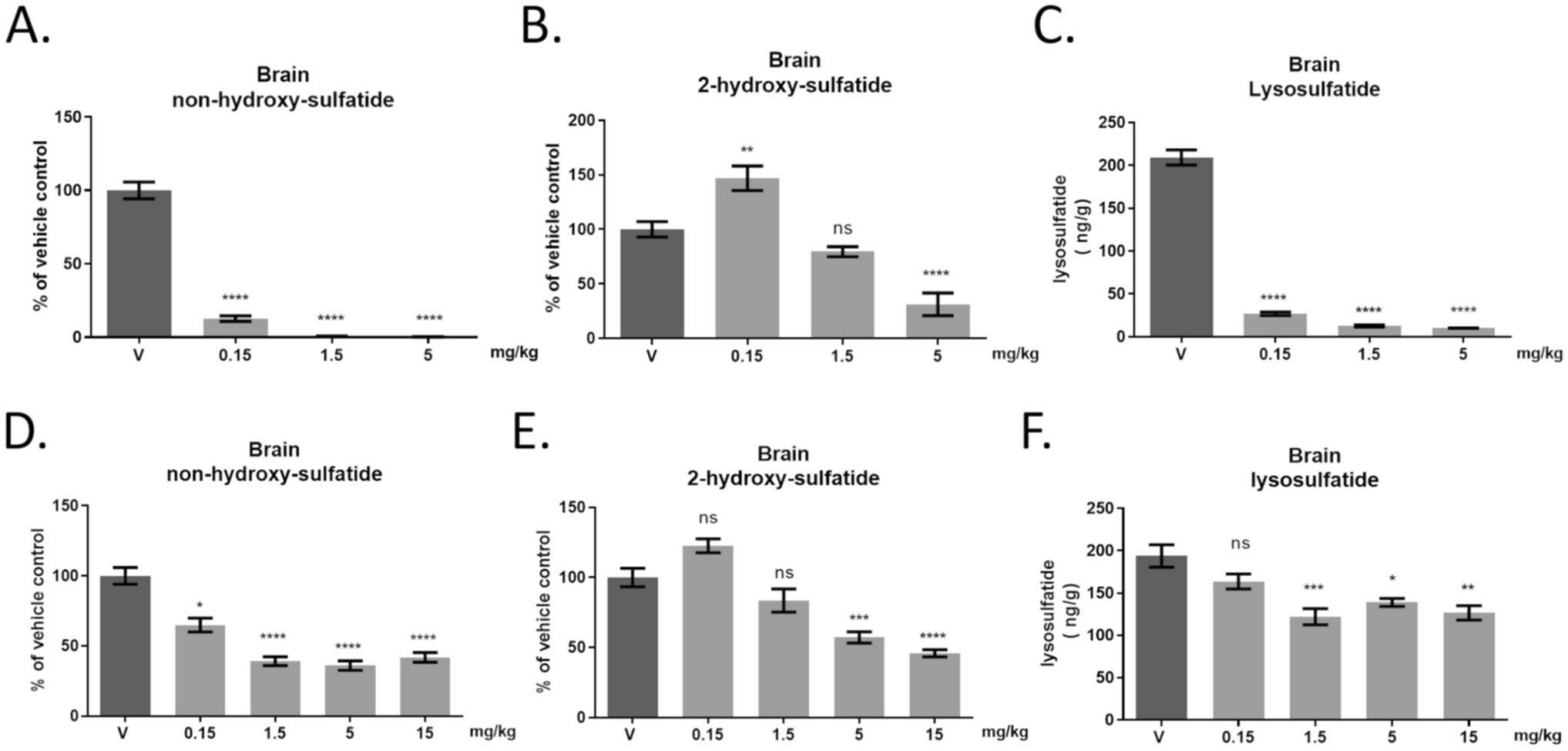
S202 treatment produced similar effects on sulfatides in an MLD mouse model. ARSA knockout mice were treated with S202 from PND3 to PND28 (n = 4, **A–C**) or PND15 to PND40 (n = 4, **D–F**). Effect of S202 treatment on non-hydroxy-sulfatide (**A,D**) and 2-hydroxy-sulfatide in brain homogenates (**B,E**). Like psychosine in Twitcher mice, lysosulfatide reduction in brain correlated with reduction of non-hydroxy-sulfatide (**C,F**).

Lysosulfatide accumulation in MLD is analogous to psychosine accumulation in KD (Fig. 1E). The ARSA-deficient mouse model of MLD has a very mild disease course; however, MLD mice do accumulate sulfatide and its cytotoxic metabolite, lysosulfatide^34,35^. Therefore, we measured the reduction of lysosulfatide following treatment with S202 IP three times per week. MLD mice treated with lower S202 doses showed a reduction in non-hydroxy-sulfatide and lysosulfatide, while higher doses reduced 2-hydroxy-sulfatides (Fig. 7A–F). Consistent with the psychosine findings, the reduction in lysosulfatide correlates with the reduction in non-hydroxy-sulfatide, suggesting acid ceramidase also prefers the non-hydroxylated form of sulfatide as a substrate. The dose-dependent changes to sulfatide levels mirrored the responses of GalCer species to treatment whether treatment started on PND3 or PND15.

### Chronic treatment of wild-type mice with S202

Due to the essential role of GalCer and sulfatides in myelin formation, we characterized the longer-term (8 week) tolerability to S202 treatment. Wild-type mice were treated IP with S202 three times per week at doses lsimilar to those that extended median Twitcher survival to ~ 60 days when treatment started on PND15 (2.3 mg/kg and 5.4 mg/kg). Generally, treatment with these dose levels were well tolerated with no mortality, change in weight, or grossly abnormal clinical signs. Clinical chemistry and hematology analysis revealed no significant effects on systemic health (see Supplementary Table S2 online). These mice also lacked histopathological changes in the major peripheral organs, with the notable exception of the testes and brain.

The testes of treated mice showed histopathological changes that were identical to those described in CGT deficient mice (see Supplementary Fig. S2 online)^36^. Specifically, the seminiferous tubules exhibited complete absence of spermatozoa and elongated spermatids, large numbers of intraluminal multinucleated giant cells, prominent germ cell exfoliation with decreased germinal epithelial layers, and the absence of residual bodies.

**Figure 8:**
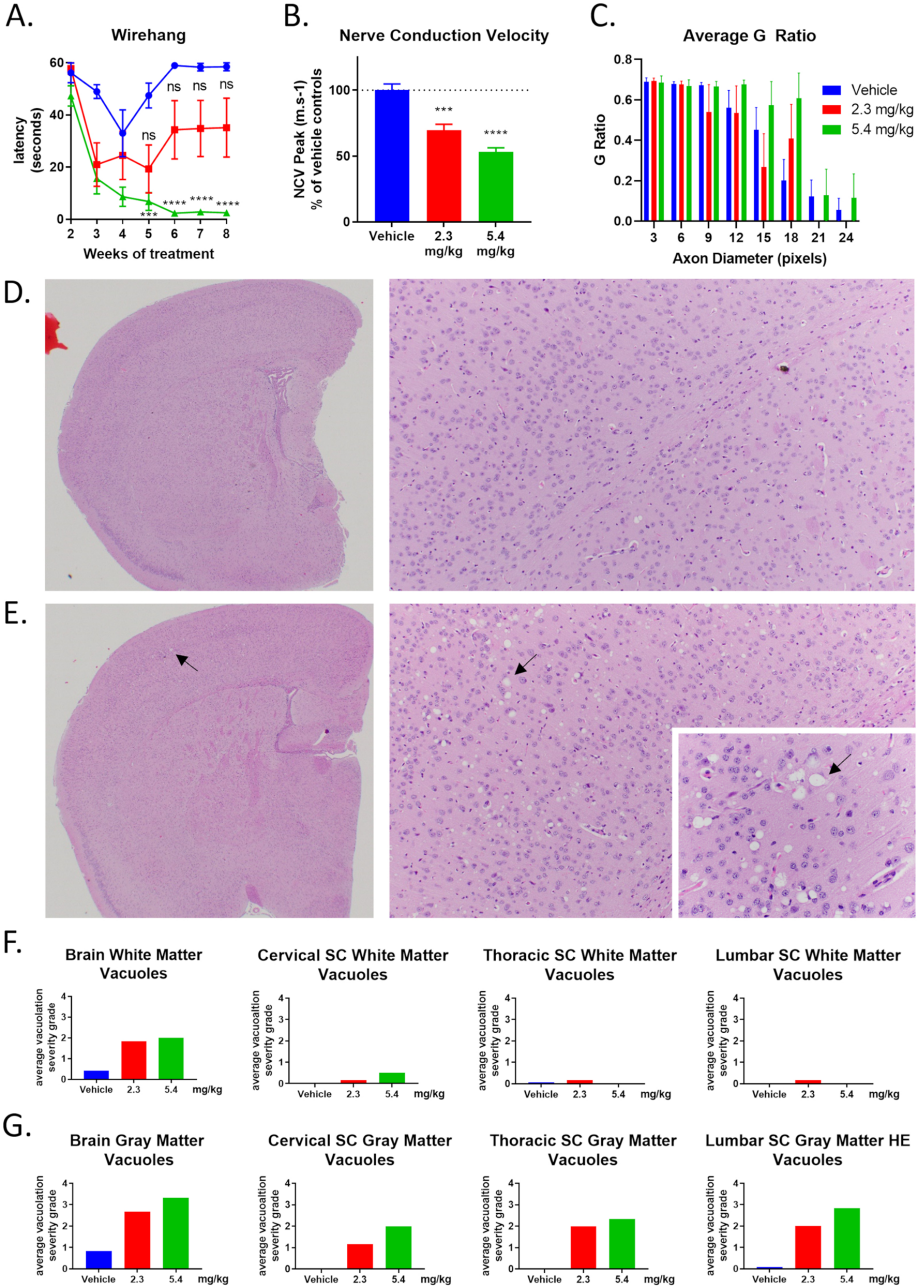
Chronic CGT inhibition affects PNS and CNS function and integrity. Wild-type mice were treated with S202 by IP injection three times per week for 8 weeks starting on PND15. The ability of mice to hang from a wire mesh was evaluated weekly starting on week 2 of treatment (**A**). Sciatic NCVs were recorded from mice that had been treated with S202 (**B**). G-ratio analysis of sciatic nerve (**C**). H&E stained brain sections from S202 revealed vacuolation in S202 treated animals at either dose (**E** arrow) that was not seen in vehicle treated animals (**D**). Severity of vacuolation was scored by a trained pathologist on a scale of 1–4 in white matter (**F**) or gray matter (**G**) in brain and spinal cord. (n = 6–12).

While seemingly well tolerated, some mice treated chronically with higher (2.3 and 5.4 mg/kg) doses of S202 developed a mild gait abnormality and impaired performance on the wire hang test (Fig. 8A). We hypothesized that these behavioral deficits resulted from CGT inhibition affecting PNS function. Therefore, nerve conduction velocity (NCV) was measured in the sciatic nerve of treated and untreated animals. There was a significant and dose-dependent reduction in NCV with treatment (Fig. 8B). Light microscopic examination of sciatic nerve did not reveal obvious structural abnormalities. However, quantitative g-ratio analysis of toluidine blue stained sections revealed a trend towards increased large diameter axons with decreased myelin thickness (Fig. 8C).

The impact of chronic S202 treatment on the CNS was evaluated by both H&E and luxol fast blue/PAS (myelin) staining of the brain and spinal cord, revealing widespread vacuolation (Fig. 8D,E). In the brain, gray matter vacuolation occurred in specific subsites, but was generally most extensive in the cerebral cortex, basal ganglia, and thalamus, with less involvement in the hippocampus, pons/medulla, and cerebellum. In all affected spinal cord segments, grey matter vacuolation was randomly scattered throughout the central area and ventral and dorsal horns. White matter vacuolation in brain/spinal cord was sporadically distributed in various subsites and characterized by variable size and sharply demarcated extracellular vacuoles scattered through the neuropil. Scoring vacuolation severity by a U.S. board certified pathologist revealed that white matter vacuolation was greater in the brain and upper spinal regions while grey matter vacuolation was more evenly distributed in all brain and spine regions (Fig. 8F,G).

The effects of longer-term CGT inhibition on GalCer levels were evaluated in the same wild-type mice. The selective reduction of non-hydroxy-GalCer and relative sparing of 2-hydroxy-GalCer levels observed in the animals receiving short-term S202 was observed in chronically treated animals (see Supplementary Fig. S3 online). We hypothesized that inhibition of CGT might also alter ceramide levels, which could negatively impact chronically treated animals. While there was only a small increase in non-hydroxy-ceramide in the animals injected with S202 (see Supplementary Fig. S3 online), there was a dramatic increase in 2-hydroxy-ceramide in S202-treated mice (see Supplementary Fig. S3 online).

In Twitcher mice treated with S202 starting at PND3, much lower doses (> 30 fold less) were needed to provide the same survival benefit observed when treatment was started at PND15. Based on these findings, we speculated that the optimal dose in younger mice was due to a balance between sufficient CGT inhibition and long-term tolerability and that this low dose level may not produce the vacuoles observed at higher dose levels. To test this hypothesis, wild-type mice were treated from PND3 to PND70 with 0.15 mg/kg S202 and brain, spine, and sciatic nerve were examined for signs of vacuolation. Surprisingly, even at this low dose, vacuolation was observed in a similar pattern, but was much less severe to that seen at higher doses (see Supplementary Fig. S4 online).

## Discussion

This study describes the identification and characterization of S202, a potent and selective inhibitor of CGT with favorable drug-like properties. While having single digit nanomolar potency toward CGT, this inhibitor only weakly inhibited the CB1 receptor (IC_50_ = 3 µM), the target of its parent analog. The favorable pharmacokinetic properties of S202 provided an opportunity to explore the consequences of CGT inhibition on myelin development and as an SRT to treat KD and MLD. These studies expand our understanding of the different roles 2-hydroxy-GalCer and non-hydroxy-GalCer play in normal development and disease progression. Finally, we also investigate the consequences of over-inhibiting CGT at different developmental stages.

Treating wild-type mice with lower doses of S202 selectively reduced non-hydroxy-GalCer synthesis with a corresponding increase in 2-hydroxy-GalCer. While the nature of this selective reduction is unknown, 2-hydroxy-Cer is a significantly more efficient substrate for CGT than non-hydroxy-Cer, possibility explaining the dose-dependent reduction of one product over the other^37^. The selective reduction of non-hydroxy-GalCer levels, while maintaining or even increasing 2-hydroxy-GalCer levels, could benefit the use of CGT inhibitors as an SRT given that 2-hydroxy forms of GalCer and sulfatide are thought to be more critical for building and sustaining the tightly packed lipid membranes of myelin sheathes^6,12^.

The ability to selectively reduce non-hydroxy-GalCer could have important therapeutic implications for KD. The reduction in non-hydroxy-GalCer correlated with the reduction in psychosine in the Twitcher mice, even while 2-hydroxy-GalCer levels were unchanged or even elevated. We also found that recombinant acid ceramidase more efficiently catabolizes non-hydroxy-GalCer to produce psychosine compared to 2-hydroxy-GalCer. Collectively, these data suggest non-hydroxy-GalCer is the primary source of psychosine and are consistent with the proportional reduction of non-hydroxy-GalCer and psychosine following CGT inhibition in vivo.

Treating neonatal Twitcher mice with S202 significantly increased survival. However, PND3 is an age in mice that precedes both CNS and PNS myelination^30^, potentially making them more sensitive to higher levels of CGT inhibition. In addition, based on developmental milestones, a PND3 mouse corresponds roughly to a third trimester human fetus^30^. Therefore, we hypothesized treatment later in development (PND15 mouse ≈ 1–2 year-old human) might be better tolerated and represent a more clinically relevant time to start treatment. When dosing was started on PND15, the tolerability to CGT inhibition greatly increased, although ~ 30-fold higher dose levels were required to achieve similar survival as when treatment started on PND3. In PND15 mice, a significant proportion of PNS myelination is complete while CNS myelination is beginning, suggesting the lower tolerability in younger mice could be from excessive CGT inhibition in the PNS.

When treatment was started on PND15, the percentage of non-hydroxy-GalCer and psychosine reduction in brain was lower compared to PND3 treatment start. Similar effects were seen for non-hydroxylated sulfatide and lysosulfatide (Fig. 7). This diminished response is likely due to preexisting non-hydroxy-GalCer, non-hydroxylated sulfatide, and/or their deacylated metabolites (psychosine and lysosulfatide) prior to treatment start on PND15. In addition, the higher doses used in PND15 mice resulted in reduction of total GalCer levels, including non-hydroxy-GalCer, psychosine, and 2-hydroxy-GalCer. These results suggest that reduction of total GalCer, not just non-hydroxy-GalCer and psychosine, contribute to survival benefit when treatment starts later in development.

Substrate reduction therapy targeting major lipid components of myelin membranes presents several challenges. These glycolipids are essential for healthy myelin formation and chronic inhibition of their synthesis could potentially destabilize myelin. Furthermore, once synthesized and incorporated into normal myelin, these molecules have half-lives approaching 1 year or more in humans^38^. With this slow turnover, SRT would need to be initiated early in development prior to GalCer and sulfatide synthesis. Alternatively, treatment would be required for several years before the steady state levels of GalCer and sulfatide, and ultimately psychosine and lysosulfatide, were significantly reduced.

Although generally well-tolerated, chronic administration of higher doses of S202 to wild-type mice resulted in dose-dependent physiological (NCV), behavioral (wire hang), and histological (vacuolation) changes. While vacuolation occurred in both grey and white matter of the spine and brain, the severity in white matter was greater in regions undergoing myelination during the treatment period and less in regions that had previously undergone significant myelination. This supports the hypothesis that tissue myelinated at treatment initiation is more resistant to adverse effects of CGT inhibition. In contrast, vacuolation observed in grey matter did not coincide with the timing of myelination and was relatively uniform in distal spine regions and brain, possibly indicating a novel role for CGT in non-myelinating cells such as astrocytes, microglia, neurons, or endothelial cells. The severity, distribution, and nature of vacuolation observed in our study is similar to CGT deficient mice as well as mice with reduced GalCer levels due to a deficiency in the *Cers2* gene^39–41^. Further studies will be needed to determine if this vacuolation is caused by altered levels of 2-hydroxy-ceramide, galactosylceramides, sulfatides or currently unknown functions of CGT in non-myelinating cells.

Although treatment of Twitcher mice with S202 resulted in significantly increased life span and decreased neuroinflammation, the improvements were modest. These results are consistent with numerous previous studies showing monotherapies result in minor therapeutic benefit in Twitcher mice. However, studies have also found dramatic and synergistic effects by combining disparate approaches that target different pathogenic mechanisms. For example, while SRT using L-cycloserine alone produces an incremental increase in survival, benefits are significantly enhanced when combined with therapies like bone marrow transplant or gene therapy. A rational combination approach might also require lower doses of a CGT inhibitor, thereby decreasing the dose-dependent toxicity observed with S202. Thus, the true potential of inhibiting CGT to treat patients with infantile KD might be observed when used in combination with other therapies. Also, juvenile and adult-onset forms of KD might respond better to CGT inhibition compared to infantile forms not only because they progress more slowly but because they also retain residual enzyme activity that can slowly metabolize lysosomal GalCer.

Ceramide galactosyltransferase inhibition reduces non-hydroxy-sulfatide and 2-hydroxy-sulfatide and could also be used to treat MLD. MLD is a less severe lipid storage disease potentially due to the lower abundance of sulfatide in myelin compared to GalCer. Because MLD is slower progressing, CGT inhibition might produce greater clinical benefits. Also, a large proportion of MLD patients are juvenile and adult-onset and could significantly benefit from lower-dose CGT inhibition. Recently, autologous stem cell gene therapy (SCGT) has resulted in significant clinical improvements in presymptomatic MLD patients^42^. Adding a small molecule CGT inhibitor to SCGT might dramatically improve the clinical outcome.

In conclusion, we have identified a novel CGT inhibitor with favorable drug-like properties that will be a valuable tool for exploring the role that GalCer and sulfatides play in normal development and disease. This inhibitor will be useful in exploring how galactosylceramides contribute to the biology of myelin, testes, and other non-myelinating cells. The ability to selectively inhibit the synthesis of non-hydroxylated galactosylceramides should be a useful attribute to enable the further understanding of the roles of the different hydroxylated species. Given that CGT inhibitors are actively being pursued as potential SRT therapy for KD and MLD^15^, further studies will be necessary to fully understand how to balance their safety and therapeutic benefit.

## Materials and methods

### Experimental design

These studies were carried out in compliance with the ARRIVE guidelines. For these unblinded, descriptive, and novel exploratory research studies, samples sizes were chosen arbitrarily and adjusted as needed. Most normal mouse, disease model, and tolerability studies were repeated independently, at least twice, at different research facilities, both academic and contract research organizations, without prior knowledge of expected outcomes. Several analogs of S202 were also tested and produced consistent findings for survival, selective biochemical changes, and tolerability. Most endpoints were determined prospectively. No data or outliers were excluded from the analysis unless noted in the methods. All methods were carried out in accordance with relevant guidelines and regulations.

### In vitro assays

#### CGT enzyme assay

Human CGT was obtained from a cell lysate from CHO cells expressing UGT8 (made by BioMarin) prepared in M-PER with proteinase inhibitors (Sigma). Compounds, serially diluted in DMSO and Tween 80, were added to 4 µg/well of CHO/CGT cell lysate to make 20 µL final reaction volume containing 0.5% DMSO and 0.01% Tween 80 in the reaction buffer consisting of 10 µM NDB-(2R-OH)-C6 ceramide (made by BioMarin), 17.5 µM UDP-Galactose (Sigma), 35 µM of dioleoylphosphatidylcholine (DOPC), 5 mM MgCl_2_, 5 mM MnCl_2_, 1% BSA, 15 mM KCl, 1 mM EGTA, 8 mM CHAPS, 10 mM HEPES–NaOH pH 7.2. The reaction was incubated at 37 °C for 1 h and stopped by adding 80 µL of methanol:acetonitrile (1:3) with 5 µM N-dodecanoyl-NBD-galactosylceramide (Matreya) internal standard (IS) for 30 min. 200 µL H_2_O:ACN (1:1) was added for 5 min and then centrifuged at 4000 rpm for 38 min at RT before quantifying NDB-(2R-OH)-C6-galactosylceramide (BioMarin) by LC–MS on a Rapid Fire 360 mass spectrometer (Agilent) coupled with a API4000 + MS in electrospray ionization (ESI) negative mode (Applied Biosystems). These assay conditions were used to screen the Bio-active lipid library (Cayman Cat# 10506).

#### Glucosylceramide synthase assay (GCS, EC 2.4.1.80)

Modified from Larsen et al.^46^. Briefly, 60 µg of MDCK cell lysate was incubated with S202 in 100 mM Tris buffer (pH 7.5), 10 mM MgCl_2_, 1 mM dithiothreitol, 1 mM EGTA, 2 mM NAD, 100 μΜ UDP-glucose, 10 μΜ C6-NBD-Ceramide (Matreya), 35 μΜ DOPC, and 5 μΜ sulfatide (Sigma) at 37 °C for 1 h. The reaction was stopped with equal volume of acetonitrile and 50 μL was added to 100 µL of acetonitrile containing 100 ng/mL tolbutamide IS and centrifuged. Next, 70 µL of supernatant was mixed with 140 μL H_2_O before LC–MS analysis. NBD-Glucosylceramide quantification was performed on a Shimadzu ultra-fast liquid chromatography system (Shimadzu) coupled with an API 4000 triple quadrupole mass spectrometer in ESI positive mode (Applied Biosystems) and a Xbridge BEH130 CI 8, 100 mm × 4.6 mm i.d, 3.5 µm column (Waters). The mobile phase consisted of water and acetonitrile supplemented with 0.1% formic acid (v/v).

#### Cerebroside sulfotransferase (CST, EC 2.8.2.11)

A 50 µL reaction consisting of 4 µg MPER lysate prepared from CHO cells stably expressing GAL3ST1 cDNA construct (made by BioMarin), 3 µM *N*-dodecanoyl-NBD-galactosylceramide or 10 µM PAPS (Sigma) added as substrate (in DMSO/Tween 80), reaction buffer (100 mM Tris/HCl (pH 7.0), 2.5 mM ATP, 20 mM MgCl_2_), and test compound. The reaction was incubated at 37 ℃ for 2 h then stopped with 100 µL of 3:1 methanol:acetonitrile (containing 500 nM NBD-C6-OH ceramide as an IS). After 1 h shaking, 150 µL 1:2 H_2_O:ACN was added, mixed, and centrifuged 4000 rpm for 30 min at RT. Supernatant was transferred for LC–MS analysis using the same conditions used for the CGT cellular assay.

#### Sphingomyelin synthase (SMS, EC 2.7.8.27)

100 µL assay buffer (50 mM Tris buffer pH 7.5 containing 25 mM KCl, 0.5 mM EDTA, 10 µM NBD-C6 ceramide, 100 µM DOPC, and 0.01% Tween 80), 60 µg MDCK cell lysate, and serial diluted compounds were incubated at 37 ℃ for 1 h. The reaction was terminated and prepared for LC–MS using the same protocol as the GCS assay, however, a NBD-C6-sphingomyelin (Avanti) reference standard was used to quantify the sphingomyelin synthesis.

#### Beta-glucocerebrosidase (GBA1, EC 3.2.1.45) & galactosylceramidase (GALC)

Recombinant human glucosylceramidase (R&D Systems) activity was monitored using the artificial fluorescent substrate 4-methylumbelliferyl-β-d-glucopyranoside (Sigma) using the assay condition on the enzyme product datasheet. Recombinant human galactosylceramidase (R&D Systems) activity was monitored using the artificial fluorescent substrate 4-methylumbelliferyl-β-d-galactopyranoside (Sigma) using the assay conditions described on the enzyme product datasheet.

#### Glucosylceramidase beta 2 (GBA2, EC 3.2.1.45) assay

A postnuclear supernatant (PNS) was prepared from HEK293 cells transiently expressing a GBA2 cDNA construct. 5 µg of PNS was added to pH 6 McIlvaine's Buffer (0.2 M Na_2_HPO_4_, 0.1 M citric acid) containing 0.2 mM 4-methylumbelliferyl-β-d-glucopyranoside substrate and serially diluted S202.

#### Arylsulfatase A (ARSA, EC 3.1.6.8)

The ARSA assay was modified from a previously reported method^47^. The reaction mixture contained 40 ng of rhARSA (R&D systems), various concentrations of inhibitor, and 6.2 µM *N*-octadecanoyl-d3-sulfatide substrate in reaction buffer (0.08 M sodium acetate at pH 4.5, 2.08 g/L sodium taurodeoxycholate, 33 mM MnCl_2_, 0.4% DMSO) and was incubated for 16 h at 37 °C. The reaction was terminated with equal volume MeOH:ethyl acetate (EA). *N*-octadecanoyl-d35-psychosine (Matreya) was added as the IS, together with 400 µL EA and 200 µL H_2_O. After centrifugation, 200 µL of the EA layer was dried under a stream of N_2_ and resuspended in 100 µL MeOH before quantifying the *N*-octadecanoyl-d3-galactosylceramide product using a reported UPLC–MS/MS method^48^.

#### UDP glucuronosyltransferase (UGT1A1, EC 2.4.1.17)

UGT1A activity was evaluated at Eurofins Panlabs Taiwan, Ltd. ITEM 196000.

#### CB1R activity

CB1R activity was evaluated at Eurofin Cerep, Le Bois l'Evêque, France ITEM G012.

#### Blood chemistry and hematology

Maximum obtainable terminal blood collection was taken via cardiac puncture and split. 200 µL into clot-activating tubes for serum processing and the following chemistry panel: AST, ALT, CKMB and electrolytes. 220 µL stored on wet ice and used for CBC.

### CGT cellular assay

3 × 10^4^ CHO cells stably expressing human UGT8 were seeded in a 96-well TC plate in F12K + 10% FBS (Invitrogen, qualified, Australia, 10099141) and incubated at 37 °C, 5% CO_2_ overnight. Media was removed and compounds, diluted in F12K + 5% FBS (0.1% DMSO final) containing 1 µM eliglustat to prevent NBD-glucosylceramide formation, were added to the cells and incubated 2 h at 37 °C. NDB-(2R-OH)-C6-ceramide (BioMarin) in F12K + 5% FBS + 11% BSA was added to a final substrate concentration of 10 µM in 1% BSA and incubated 1 h at 37 °C before extracting in methanol/0.5% acetic acid containing *N*-dodecanoyl-NBD-galactosylceramide IS (1 µM). After 2 h shaking, the solution was centrifuged at 4000 rpm for 30 min, and 80 µL of supernatant was transferred to 40 µL H_2_O, mixed and centrifuged at 4000 rpm for 10 min. NDB-(2R-OH)-C6-galactosylceramide product was quantified by a Shimadzu ultra-fast liquid chromatography system (Shimadzu) coupled to an API 4000 mass spectrometer in ESI negative mode (Applied Biosystems) using a Kinetex C18 column (50 × 2.1 mm, 2.6 µm, 100 Å) (Phenomenex) with 0.1% formic acid and acetonitrile supplemented with 0.1% formic acid.

#### In vitro psychosine production

25 µM of 2-hydroxy-GalCer or non-hydroxy-GalCer (Matreya) were incubated with 7 µL of purified acid ceramidase in a reaction containing 15 µL of 0.2 M citrate phosphate buffer (pH 4.5), 2.25 µL of 2 M NaCl, 1.5 µL of 10 mg/mL BSA, and 0.3 µL 10% IGEPAL CA630. The reaction was incubated at 37 °C for 18 h without agitation and then stopped by adding 60 µL of acidified methanol. Psychosine formation was determined by LC–MS. Briefly, samples were extracted in acetonitrile containing d5-glucosysphingosine (Avanti) IS and analyzed using a Shimadzu HPLC/Autosampler with Halo-Hilic (150 mm, 5 µm) and an API-4000 Qtrap Mass Spectrometer (ESI positive, multiple reaction monitoring [MRM] scan). Mobile phase A was 0.5% FA, 5 mM NH_4_Ac in water. Mobile phase B was ACN/MeOH/0.5% FA 5 mM NH_4_Ac; (95/2.5/2.5). Samples were quantified using a psychosine (Avanti) standard curve.

### Mouse studies

All procedures were carried out in accordance with approved Institutional Animal Care and Use Committee (IACUC) protocols and the BioMarin Animal Resource Committee (ARC). Twitcher mouse studies were conducted using a protocol approved by the Washington University School of Medicine IACUC. PND3 wild-type mouse studies and ARSA -/- studies were conducted using a protocol that was approved by the Shanghai ChemPartner Co. IACUC. PND15 wild-type mice tolerability studies were conducted using a protocol approved by the Jackson labs IACUC. For all animal studies S202 was formulated in 5% dimethylacetamide (Sigma-Aldrich), 10% Solutol HS15 (Sigma-Aldrich), 85% 10 mM citrate buffer (pH 4.5).

#### Pharmacokinetics

C57BL/6 mice were given a single 20 mg/kg IP injection of S202 and plasma pharmacokinetics and brain tissue levels were monitored (n = 3 per timepoint). Blood was collected in K_2_ EDTA tube to obtain a plasma sample (2000*g*, 5 min at 4 °C). The brain was removed, rinsed with cold saline, dried on filter paper, weighed, and snap frozen by placing on dry ice. Brains were homogenized in 3 volumes of PBS (pH 7.4) by mini-bead-beater before sample extraction for bioanalysis. A UPLC-MS/MS-22 (6500) was used to quantify S202 levels relative to a Diclofenac IS and S202 standard curve. Pharmacokinetic parameters were calculated using WinNonlin V 8.2 statistics software (Pharsight Corporation).

#### PND3 wild-type mouse repeat dose studies

Wild-type C57BL/6 mice were given daily IP injections of S202 from PND3 to PND40 and weight and clinical signs were monitored daily. Animals were euthanized by exsanguinations under deep anesthesia, the brain and sciatic nerve were removed, rinsed in cold saline, dried, and snap frozen for lipid quantification.

#### Twitcher mouse studies

Mice heterozygous for the Twitcher mutation on the congenic C57BL/6 background were originally purchased from The Jackson Laboratories (stock #000845). Homozygous GALC −/− animals were generated from heterozygous mating. Genotypes were determined on PND1 by PCR as previously described^43,44^. Animals that died prior to weaning on PND28 were excluded from the study unless otherwise noted. Animals were euthanized when they became moribund as defined by any of the following: losing 25% of their maximal body weight, ataxia severe enough to impair ability to eat or drink, or lack of response to tactile stimulus. Animals used for tissue collection were euthanized by anesthetic overdose and perfused transcardially with PBS for three min. Animals for lifespan analysis were euthanized using CO_2_ compressed gas delivered at 10–30% chamber volume displacement per min. Treatment IP with S202 began on either PND3-4 or PND15-16 and continued three times/week for the life of the animal. The thrice weekly dosing schedule was selected based on the long in vivo half-life of S202 and to simplify the dosing schedule. Tissues were harvested and flash frozen in liquid nitrogen.

#### ARSA knockout mice (PND3 and PND15)

ARSA mice were obtained from Volkmar Gieselmann, University of Bonn. ARSA −/− mice were generated from heterozygous pairing and confirmed by PCR genotyping. Treatment IP with S202 began on either PND3 or PND15 and continued until PND28 or PND40 respectively. Animals were euthanized by exsanguination under deep anesthesia and brain and sciatic nerve removed, rinsed in cold saline, dried, and snap frozen for lipid quantification.

#### PND15 wild-type mice tolerability studies

Treatment IP with S202 began on PND15-16 and continued three times/week until PND71. Animals were euthanized with CO_2_ followed by cardiac puncture. Blood was collected via cardiac puncture and split for blood chemistry and hematology.

#### Wire hang assay

Beginning on the 2nd week of treatment mice were trained weekly for two weeks. Mice are placed on a wire grid, which is shaken to increase wire grabbing, and then immediately turned over. Mice are timed for their ability to hang from the inverted grid for 1 min. Three trials are performed, up to 1 min each, with a 30 s recovery period between trials. After training, mice were tested weekly and times recorded. The test was done with the grid held 10–12 in. over a box of clean shavings.

### Young wild-type and MLD mouse galactosylceramide and sulfatide analysis

#### Non-hydroxy-GalCer and 2-hydroxy-GalCer

Whole brain was homogenized in 3 volumes H_2_O (v/w) and further diluted as necessary in H_2_O. Given the small size, the sciatic nerve weight was recorded and homogenized in a larger fixed volume. 25 µL of diluted homogenate was added to 35 µL DMSO, 200 µL of methanol, and 325 µL acetone/methanol (50/50) and vortexed approximately 30 min. Next, 150 µL water and 300 µL water/methanol (13/87) were added before centrifuging for 5 min at RT at 14,000 rpm. 1200 µL supernatant was transferred onto a pre-conditioned C18-SPE cartridge, washed with 2 mL methanol/acetone/water (67/23/10), and eluted with 1.5 mL acetone/methanol (90/10). The eluate was dried before reconstituting with 50 µL DMSO (including N-C16:0-CD3-Glucosylceramide, IS Matreya) and 200 ACN. 3 µL of suspension was injected into LC–MS/MS for analysis. GalCers were separated from isobaric GluCers by Halo-Hilic (4.6 × 150 mm, 2.7 µm) column with a mobile phase of 95/2.5/2.5 ACN/MeOH/H_2_O (with 0.5 FA + 5 mM NH_4_OAc) 100% for 9.5 min at 50 °C and a 0.50 mL/min flow rate. Non-hydroxy-GalCer and 2-hydroxy-GalCer were detected using MRM on an LC–MS/MS (Agilent 6410) in positive ESI mode. Purified non-hydroxy-GalCer (Kerasin, Matreya) or 2-hydroxy-GalCer (Phrenosin, Matreya) were used as reference standards. All non-hydroxy-GalCer or 2-hydroxy-GalCer species identified in the reference standards were summed, normalized to the IS, and reported as percentage of vehicle control (Fig. 2B,C). In Supplementary Figure S1, individual acyl chains were identified, normalized to the IS, and plotted as percentage of vehicle control.

#### Non-hydroxy-sulfatide and 2-hydroxy-sulfatide

Previously diluted brain homogenate was serially diluted (10 µL into 90 µL and then 20 µL into 80 µL) in ACN/H_2_O (v/v 1:1) before being added to 300 µL ACN with IS (N-C18:0-CD3-sulfatide, Matreya). The mixture was vortexed for 10 min, centrifuged at 5800 rpm for 10 min, and then 3 µL of supernatant was injected for LC–MS/MS analysis. Sulfatides were separated on an ACE 5 C18 (2.1 × 50 mm, 5 µm) column (0.6 mL/min flow rate) at 60 °C using mobile phase A (H_2_O-5 mM NH_4_OAc) and mobile phase B (ACN). Non-hydroxy-sulfatide and 2-hydroxy-sulfatide were detected using MRM on a UPLC-MS/MS-22 (Triple Quad 6500) in positive ESI mode. Total sulfatides (Matreya) were used as reference standards. The non-hydroxy-sulfatide or 2-hydroxy-sulfatide species identified in the reference standards were each summed and normalized relative to the vehicle treated animals.

#### Twitcher psychosine and galactosylceramide analysis

Galactosylsphingosine (psychosine), and galactosylceramide were measured by tandem mass spectrometry as previously described^45^. Briefly, tissue samples were homogenized in 0.04 M citric acid. Internal controls in 200 µL methanol were added to 50 µL of each sample. Galactosylsphingosine and galactosylceramide were separated from glucosylsphingosine and glucosylceramide by HILIC columns. Galactosylsphingosine and galactosylceramide were detected using MRM on an AB SCIEX 4000QTRAP tandem mass spectrometer using ESI in the positive ion mode. Data were processed with Analyst 1.5.2 (Applied Biosystems). Data are reported as the peak area ratios of lipids to their ISs.

#### ARSA knockout lysosulfatide and sulfatide analysis

Sulfatides were quantified using the methods described above. For lysosulfatide, 30 µL of brain homogenates were added to 200 µL ACN with IS (*N*-acetylsulfatide, Matreya), vortexed for 10 min, and centrifuged at 5800 rpm for 10 min. 3 µL of supernatant was injected for LC–MS/MS analysis. UPLC–MS/MS methods were the same used for sulfatides described above except lysosulfatide (Matreya) was used as a reference standard.

#### P15 wild-type mouse brain and sciatic nerve galactosylceramide analysis

The right midsagittal brain hemisphere was harvested, wet weights recorded, and then homogenized in 1 mL and sciatic nerve in 300 µL of H_2_O using a Bead Ruptor Bead Mill Homogenizer (Omni International). Proteins were quantified using the BCA Assay kit (Thermo Fisher Scientific) as described in the provided protocol and analyzed using a Tecan Infinite M1000 plate reader. Brain homogenate was diluted to 4 µg protein/µL and sciatic nerve was diluted to 0.25 µg protein/µL in water. 50 µL of homogenate was mixed with 500 µL of acidified methanol (95% methanol, 5% glacial acetic acid v/v) and incubated at room temperature for 2 h with intermittent vortexing (every 15–20 min). Extracted samples were spun for 5 min at 5000 rpm, then supernatants transferred to 10 kDa centrifugal filters, and centrifuged at 12,000 rpm for 30 min at room temperature. Ceramides and galactosylceramides were analyzed by LC–MS/MS (Waters UPLC Xevo TQ-S) using a Waters Acquity UPLC BEH C8 Column (130 Å, 1.7 µm, 1.0 mm × 150 mm) with mobile phase A (74% methanol, 25% H_2_O, 1% formic acid, 5 mM ammonium formate) and mobile phase B (99% methanol, 1% formic acid, 5 mM ammonium formate). With a column temperature of 50 °C and a flow rate of 0.07 mL/min, the initial 20% B was ramped up to 100% B in 12.0 min, maintained until 18.0 min, returned to 20% B at 18.1 min, and sustained until 20.0 min. Quantification was done based on a standard curve using GalCer(24:1), Cer(24:1) and Cer(24:1)2R-OH (Avanti). Hydroxy-galactosylceramide was quantified using the non-hydroxylated standard as a proxy. Quantified ceramides are expressed as pmol per microgram of total protein.

#### Twitcher mouse immunostaining

Five sections from a one in four series of sagittal brain sections from each mouse were stained on slides using a modified immunofluorescence protocol for the following antibodies—astrocytes (rabbit anti-GFAP, 1:1000, DAKO Z0334), microglia (rat anti-mouse CD68, 1:400, Bio-Rad MCA1957), and myelin basic protein (MBP) (rat anti-MBP, 1:500, Merck Millipore MAB386). Briefly, 40 µm sagittal sections were mounted on *Superfrost Plus* slides (Fisher Scientific), air-dried for 30 min, and blocked in 15% normal goat serum solution (Vector Labs, S-1000) in 2% TBS-T (1 × Tris Buffered Saline, pH 7.6 with 2% Triton-X100, Fisher Scientific) for 1 h. Slides were then incubated in primary antibody in 10% serum solution in 2% TBS-T for 2 h. Slides were washed three times in 1×TBS and incubated in fluorescent Alexa-Fluor labelled IgG secondary antibodies (Alexa-Fluor goat anti-rabbit 488, Invitrogen A-11008 and goat anti-rat 546, Invitrogen A-11081) in 10% serum solution in 2% TBS-T for 2 h, washed three times in 1×TBS, and incubated in a 1 × solution of *TrueBlack* lipofuscin autofluorescence quencher (Biotium, Fremont, CA, #23,007) in 70% ethanol for 2 min before rinsing in 1×TBS. Slides were coverslipped in fluoromount-G mounting medium with DAPI (Southern Biotech). To analyze the glial activation (GFAP-positive astrocytes + CD68-positive microglia) as well as MBP staining of punctate bodies in the cerebellar white matter, a semiautomated thresholding image analysis method was used with *Image-Pro Premier* software (Media Cybernetics). Briefly, this involved the collection of slide-scanned images at 5 × magnification for a one in four series of 3 sections per animal followed by demarcation of all regions (in this case the white matter of the cerebellum) of interest while maintaining the lamp intensity, video camera setup, and calibration constant throughout image capturing. Images were subsequently analyzed using *Image-Pro Premier* using an appropriate threshold that selected the foreground immunoreactivity above background. This threshold was then applied as a constant to all subsequent images analyzed per batch of animals and reagent used to determine the specific area of immunoreactivity for each antigen. Image densitometry for the average pixel luminance data was also gathered for MBP staining of the cerebellar white matter due to the higher density of staining. Slide-scanned images at 5 × magnification were collected for a one in forty-eight series of sections per animal followed by collecting the mean luminance data across all pixels from the regions of interest delineated using *Image-Pro Premier.*

#### Cytokine/chemokine analysis

Cytokine/chemokine analyses were performed on the brains from the same animals used for immunofluorescent imaging. Briefly, one sagittal half of the brain was homogenized in 10 mM Tris (pH 7.5), 150 mM NaCl, 0.1% Triton X-100, and 2 mM EDTA containing a protease inhibitor cocktail (Sigma, St. Louis, MO, cat# p8849). The lysates were adjusted to 10 mg/mL total protein with PBS, centrifuged at 10,000 g × 10 min 4 °C to remove particulates, then 25 µL per well in duplicate was added to 25 µL PBS with premixed magnetic beads (ThermoFisher/Procartaplex mouse simplex beads) capturing IL-10, MIP-1α, GRO-α, MCP-1, IP-10, IL-1β, IFNγ, IL-12p70, IL-4, IL-5, IL-6, and TNF-α. The plate was incubated overnight on a shaker at 4 °C with the final detection steps/analysis using the Luminex FlexMAP3D performed the next morning. A calibration curve was generated from the manufacturer-expressed-protein standard using a 5-parameter curve fitting software program (Milliplex Analyst 5.1, Verigene) and a two-fold dilution factor applied to the extrapolated pg/mL sample values.

#### WT mouse tolerability study histology, morphometric analysis, and vacuole scoring

Brains were collected after removal of the calvarium, weighted, and halved following the midline. The brain was sectioned in the mid-sagittal plane with half placed in 10% NBF. One sciatic nerve and other organs [heart, lung, liver, spleen, kidney, adrenal glands, stomach, small intestine, large intestine, spinal cord (cervical, thoracic, lumbar), skin, testes, and lymph nodes] were also collected and fixed in 10% NBF. Sections of sciatic nerve were also fixed in 2% PFA, 2% glutaraldehyde, embedded, sectioned and stained with Toluidine blue before scanning. Images were analyzed to determine the G-ratio in the dorsal funiculus.

#### NCV

Mice were anesthetized with isoflurane and placed on a thermostatically controlled heating pad to maintain body temperature. The recording electrode was inserted into the plantar muscles and a reference electrode was placed in the skin between toes. A pair of subcutaneous stimulating electrodes were placed distally on both sides of the ankle to stimulate the lateral plantar nerve. For proximal stimulation, the electrodes are moved to the level of the sciatic notch, to the depth of the sciatic nerve. A current pulse was delivered at low frequency and was gradually increased from zero until a maximal compound muscle action potential (CMAP) in the plantar muscles was evident on the oscilloscope. Six distally-produced CMAPs were recorded and stored. The procedure was repeated using the proximal electrode pair, and current was increased until a CMAP that matches that produced by distal stimulation was evident. Six proximally-produced CMAPs were recorded and stored. The distance between the sites of stimulation was measured. NCV was later calculated using the two latencies and conduction distance. The H wave was recorded during the recording of the distally stimulated CMAPs.

### Statistical analysis

Significance from control group are represented as follows: * = P ≤ 0.05, ** = P ≤ 0.01, *** = P ≤ 0.001, **** = P ≤ 0.0001. All error bars represent standard error. A two-way repeated measures ANOVA with Tukey correction for multiple comparisons was used for weight curves and wire hang. All glycolipid bar graphs, immunohistochemistry, and cytokine analyses used one-way ANOVA on log-transformed data with Tukey correction for multiple comparisons. Log-rank (Mantel-Cox) test with Bonferroni correction was used for survival curves. In vitro psychosine generation used an unpaired t-test. IC_50_ values were generated from sigmoidal dose–response (variable slope) curves. Values that were below the limit of quantitation for various bioanalytical studies were labeled BLQ and excluded from statistical analysis of log transformed data, with the exception of MCP-1.

## Supplementary Information

Supplementary Information.

## Data Availability

S202 may be obtained through an MTA with BioMarin.
