# Supplementary material for: Substrate reduction therapy for Krabbe disease and metachromatic leukodystrophy using a novel ceramide galactosyltransferase inhibitor
Source: Sci Rep. 2021 Jul 14;11:14486. doi: 10.1038/s41598-021-93601-1 (PMC8280112; doi:10.1038/s41598-021-93601-1)
Supplement: Supplementary file 1 — Supplementary Information. [file 41598_2021_93601_MOESM1_ESM.pdf]

Supplemental Information for:

Substrate Reduction Therapy for Krabbe Disease and Metachromatic Leukodystrophy using a Novel Ceramide Galactosyltransferase Inhibitor.

Michael C. Babcock[1+], Christina R. Mikulka[2+], Bing Wang[1], Sanjay Chandriani[1], Sundeep Chandra[1], Yue Xu[1], Katherine Webster[1], Ying Feng[1], Hemanth R. Nelvagal[4], Alex Giaramita[1], Bryan K. Yip[1], Melanie Lo[1], Xuntian Jiang[2], Qi Chao[1], Josh C. Woloszynek[1], Yuqiao Shen[1], Shripad Bhagwat[1], Mark S. Sands[2,3], Brett E. Crawford[1*]

*To whom correspondence should be addressed: Brett E. Crawford, 105 Digital Drive, Novato, CA 94949, bcrawford@bmrn.com.

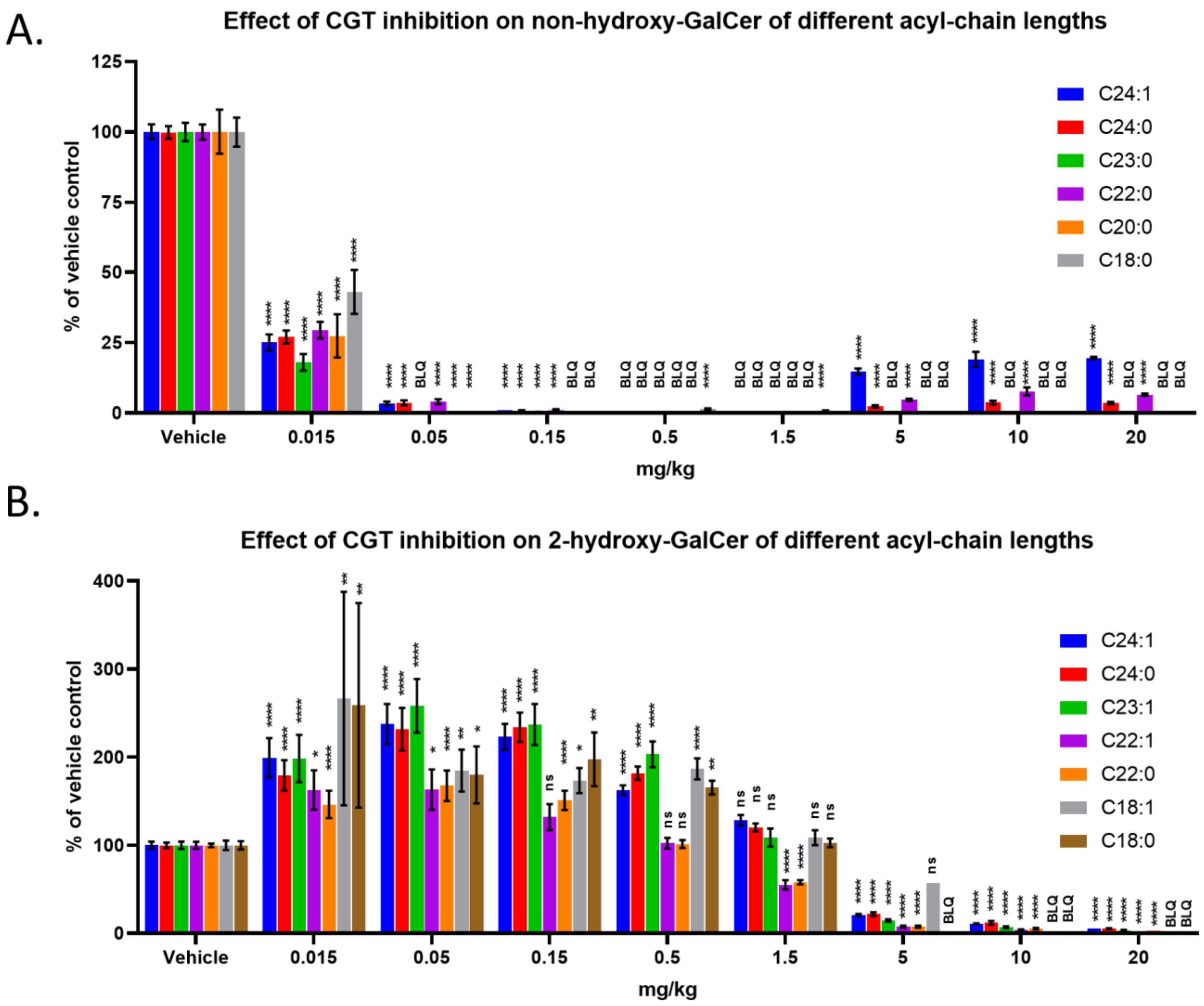

Supplementary Figure S1. Response of individual acyl chains to CGT inhibition *in vivo*. LC-MS quantification of individual acyl chain forms of non-hydroxy-GalCer (**A**) and 2-hydroxy-GalCer (**B**) in brain from mice treated with S202 from PND3-PND40 (n=2-17).

A. B.

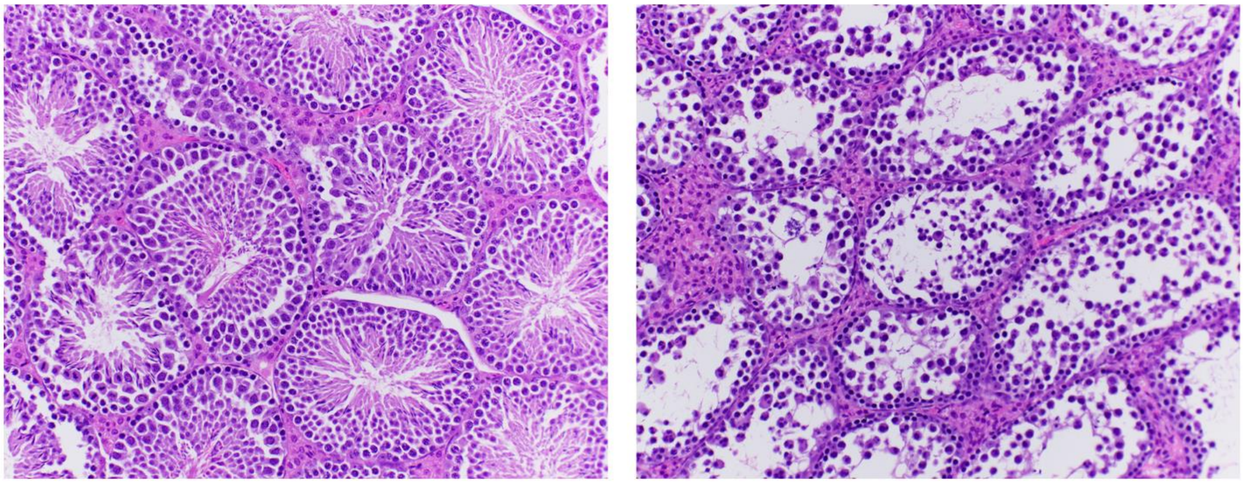

Supplementary Figure S2. Impact of S202 on testes. Testes of wild-type mice treated with S202 from PND15 to PND70. Compared to vehicle treated mice (**A**), the testes of S202 treated mice (**B**) showed seminiferous tubules with complete absence of spermatozoa and elongated spermatids, large numbers of intraluminal multinucleated giant cells, germ cell exfoliation with decreased germinal epithelial layers, and complete absence of residual bodies.

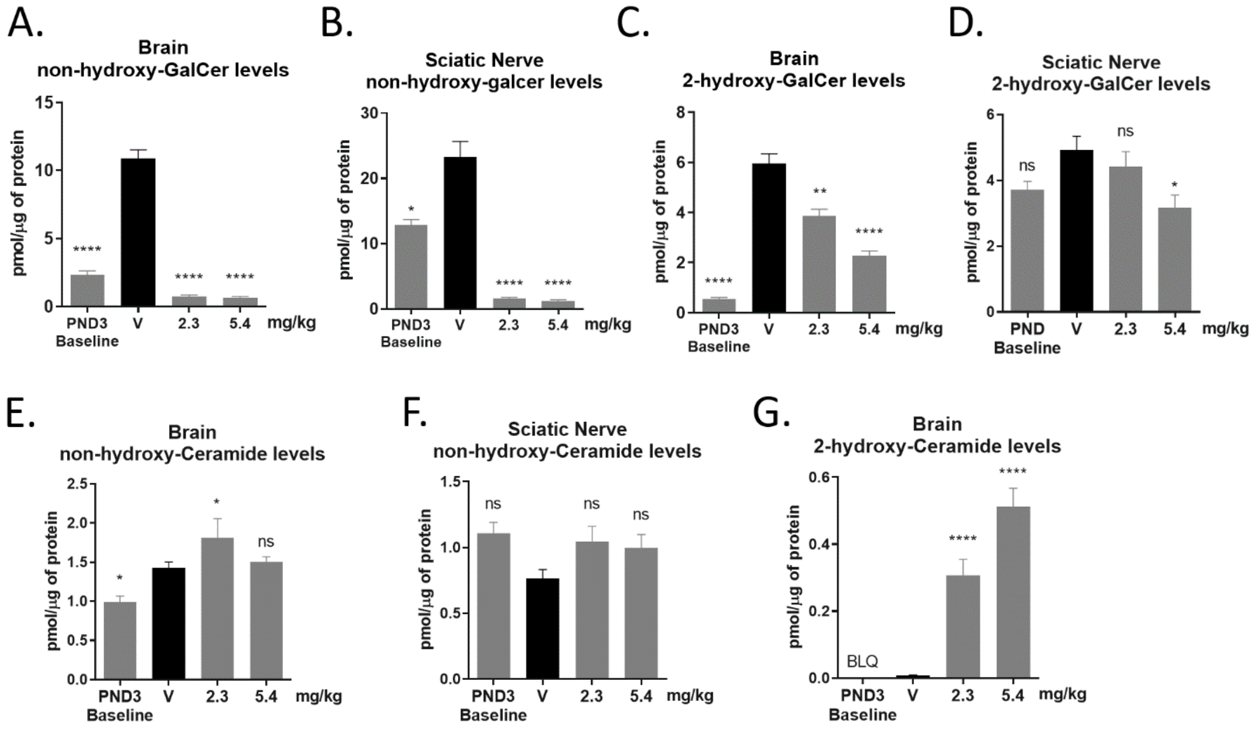

Supplementary Figure S3. Impact of S202 on glycosphingolipids in wild-type mice treated from PND15-70.

Non-hydroxy-GalCer were reduced in brain (**A**) and sciatic nerve (**B**) in treated mice while 2-hydroxy-GalCer were less reduced in brain (**C**) and sciatic nerve (**D**). Non-hydroxy-ceramide levels were not significantly altered in brain and sciatic nerve from treated mice (**E and F**). However, 2-hydroxy-ceramide levels in brain were greatly increased in treated mice (**G**). (n=6-12).

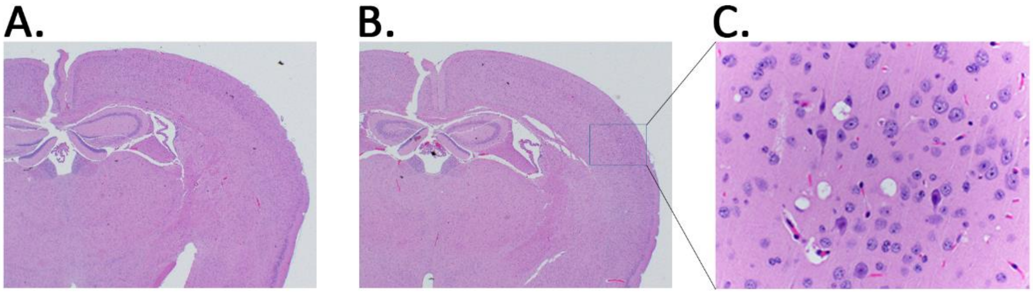

Supplementary Figure S4. Low dose treatment (0.15 mg/kg) starting on PND3-70 produces similar brain vacuolation.

Wild-type mice were treated from PND3 to PND70 with vehicle (**A**) or 0.15 mg/kg S202 (**B and C**) and brains evaluated for the presence of vacuoles. Vacuoles were found in the same regions as observed at higher doses when treatment was started on PND15.

Enzymes tested	S202 IC50
CGT enzyme	15 nM
CGT cellular	3 nM
GCS	>50 µM
GBA1	>50 µM
GBA2	>50 µM
GALC	>50 µM
CST	>50 µM
ARSA	>50 µM
SMS	>50 µM
UGT1A	>30 µM
CB1R inverse agonist	2.1 µM
CB1R antagonist	2.9 µM

Supplementary Table S1. S202 CGT inhibition selectivity.

S202 inhibitor activity against enzymes with related substrates. Activity towards the CB1 receptor was also evaluated.

	Vehicle		S202 2.3 mg/kg			S202 5.4 mg/kg		
	Avg	SD	Avg	SD	P value	Avg	SD	P value
ALT IU/L	84.8	70.5	257.2	169.5	0.008	99.5	67.2	0.96
AST IU/L	155.7	74.2	283.5	106.8	0.010	132.8	47.9	0.83
CKMB U/L	197.4	44.0	250.5	98.8	0.21	126.4	36.7	0.071
NA mmol/L	146.4	3.4	148	2.0	0.53	150.9	2.6	0.015
K mmol/L	9.9	1.1	10.5	1.3	0.45	9.6	0.6	0.84
CL mmol/L	107.8	1.9	109.4	3.8	0.44	111.3	2.0	0.031
WBC x10^3/µL	5.7	1.2	5.5	0.7	0.93	4.9	0.3	0.26
RBC x10^6/µL	10.4	0.5	9.9	0.3	0.41	10.4	0.4	>0.99
HGB g/dL	15.2	1.0	15.3	0.4	0.99	15.9	0.4	0.22
HGBCell g/dL	13.4	0.5	13.2	0.2	0.78	13.7	0.4	0.53
HCT %	50.1	2.1	47.9	0.9	0.34	50.1	1.7	>0.99
MCV fL	48.2	0.9	48.2	0.5	>0.99	48.1	0.5	0.98
MCH pg	14.6	0.8	15.4	0.1	0.28	15.3	0.2	0.13
MCHC g/dL	30.3	1.8	31.9	0.1	0.32	31.8	0.5	0.14
CH pg	12.9	0.3	13.3	0.2	0.19	13.1	0.2	0.45
CHCM g/dL	26.7	0.2	27.5	0.1	0.03	27.3	0.4	0.024
RDW %	12.7	0.4	12.2	0.4	0.11	11.8	0.3	0.0012
HDW g/dL	1.91	0.05	1.9	0	0.71	1.93	0.03	0.69
PLT x10^3/µL	817.3	258	1020.0	41	0.63	737.2	298	0.86
PDW %	42.83	8.0	37.1	1.3	0.78	46.63	13.3	0.81
MPV fL	5.5	0.21	5.4	0.1	0.82	5.82	0.39	0.21
MPM pg	1.31	0.02	1.3	0.0	0.93	1.3	0.02	0.59
%NEUT	16.9	6.8	14.3	1.9	0.84	11.4	5	0.26
%LYMPH	72.5	8.4	78.8	3.4	0.62	77.6	8.5	0.55
%MONO	3.0	1.1	2.3	0.6	0.53	1.6	0.6	0.040
%EOS	5.8	2.6	2.4	1.0	0.65	8.2	6.2	0.64
%BASO	0.4	0.14	0.4	0.0	>0.99	0.35	0.12	0.78
%LUC	1.3	0.5	2	1.1	0.36	0.9	0.4	0.46
%RETIC	3.2	0.4	2.9	0.8	0.63	2.7	0.3	0.13
#RETIC 10^9cells/L	335	52.3	291	90.2	0.51	285	23.7	0.20
CHR pg	15.1	0.6	14.6	0.1	0.24	14.6	0.1	0.10
CHm pg	13.6	0.3	14	0.2	0.24	13.8	0.2	0.47
#NEUT 10^3cells/µL	0.92	0.26	0.8	0.2	0.77	0.55	0.24	0.06
#LYMPH 10^3cells/µL	4.2	1.2	4.3	0.3	0.99	3.8	0.5	0.69
#MONO 10^3cells/µL	0.18	0.07	0.12	0.01	0.38	0.08	0.03	0.02
#EOS 10^3cells/µL	0.32	0.11	0.14	0.08	0.59	0.4	0.31	0.81
#BASO 10^3cells/µL	0.022	0.012	0.02	0.0	0.97	0.013	0.005	0.26
#LUC 10^3cells/µL	0.073	0.031	0.11	0.07	0.40	0.047	0.021	0.38

Supplementary Table S2. Clinical blood chemistry and blood cell counts for CGT inhibitor treated wild-type mice.

Clinical chemistry and hematology from wild-type mice treated from PND15-PND70. For CBC n= 12 for vehicle and n= 6 for low and high dose groups. For blood cell counts n=6 for vehicle and high dose group but n=2 for low dose group. Statistics are 1-way ANOVA with Tukey post hoc analysis.
